# North-south cross sections of the vertical aerosol distribution over the Atlantic Ocean from multiwavelength Raman/polarization lidar during Polarstern cruises

**DOI:** 10.1002/jgrd.50273

**Published:** 2013-03-26

**Authors:** T Kanitz, A Ansmann, R Engelmann, D Althausen

**Affiliations:** 1 Leibniz Institute for Tropospheric ResearchLeipzig, Germany

## Abstract

Shipborne aerosol lidar observations were performed aboard the research vessel Polarstern in 2009 and 2010 during three north-south cruises from about 50°N to 50°S. The aerosol data set provides an excellent opportunity to characterize and contrast the vertical aerosol distribution over the Atlantic Ocean in the polluted northern and relatively clean southern hemisphere. Three case studies, an observed pure Saharan dust plume, a Patagonian dust plume east of South America, and a case of a mixed dust/smoke plume west of Central Africa are exemplarily shown and discussed by means of their optical properties. The meridional transatlantic cruises were used to determine the latitudinal cross section of the aerosol optical thickness (AOT). Profiles of particle backscatter and extinction coefficients are presented as mean profiles for latitudinal belts to contrast northern- and southern-hemispheric aerosol loads and optical effects. Results of lidar observations at Punta Arenas (53°S), Chile, and Stellenbosch (34°S), South Africa, are shown and confirm the lower frequency of occurrence of free-tropospheric aerosol in the southern hemisphere than in the northern hemisphere. The maximum latitudinal mean AOT of 0.27 was found in the northern tropics (0– 15°N) in the Saharan outflow region. Marine AOT is typically 0.05 ± 0.03. Particle optical properties are presented separately for the marine boundary layer and the free troposphere. Concerning the contrast between the anthropogenically influenced midlatitudinal aerosol conditions in the 30– 60°N belt and the respective belt in the southern hemisphere over the remote Atlantic, it is found that the AOT and extinction coefficients for the vertical column from 0–5km (total aerosol column) and 1–5km height (lofted aerosol above the marine boundary layer) are a factor of 1.6 and 2 higher at northern midlatitudes than at respective southern midlatitudes, and a factor of 2.5 higher than at the clean marine southern-hemispheric site of Punta Arenas. The strong contrast is confined to the lowermost 3km of the atmosphere.

## 1. Introduction

Atmospheric aerosols influence the radiation budget of the Earth and the water cycle via aerosol-cloud interactions. The impact is especially sensitive over the oceans [Stevens and Feingold, 2009]. For instance, investigations suggest a potential aerosol impact on the strengths of hurricanes [Rosenfeld *et al.* 2011]. Gaps in our knowledge regarding the vertical distribution of aerosols around the world, in particular over the oceans, and the variability of aerosols in terms of particle concentration, type, and mixing state, lead to discrepancies between results of different global aerosol transport models [*Textor et al*., 2006]. An improved consideration of aerosol effects in climate models can only be achieved by extending the observation and characterization of aerosol layers towards the relevant regions. The vast, hardly accessible oceans embodied a poorly characterized area in atmospheric science until the end of the 1960s. First investigations were based on in situ observations within the marine boundary layer (MBL) [*Fitzgerald*, 1991; *Heintzenberg et al.* 2000; *Smirnov et al.* 2002]. A systematic global mapping of aerosols above the oceans started with the first spaceborne aerosol observations in the 1970s [*Prospero et al.* 2002]. In the 1990s, a variety of satellite sensors was developed and permitted an improved particle characterization on a global scale. The mean aerosol optical thickness (AOT) at 550-nm wavelength was determined by the Moderate Resolution Imaging Spectroradiometer (MODIS), the Sea-viewing Wide field-of-view sensor (SeaWifs), and the Advanced Very High Resolution Radiometer (AVHRR-2) to be in the range of 0.10–0.16 above the oceans [*Remer et al.* 2008; *Myhre et al.* 2005].

Continuous ground-based aerosol observations have been performed at single islands in the framework of Aerosol Robotic Network (AERONET) activities since June 1994, when observations were started at Mauna Loa, Hawaii [Holben *et al.* 2001]. A comparison between observations on islands showed values of AOT at 500nm of 0.13–0.14 and 0.07–0.08 over the Atlantic and the Pacific Ocean, respectively [*Smirnov et al.* 2002]. Large progress was achieved with the introduction of the handheld Microtops Sun photometer observations that are used aboard research vessels (RV) to determine the AOT above the oceans in the framework of Maritime Aerosol Network (MAN) since October 2004 [*Smirnov et al*., 2009, 2011]. These observations revealed the lowest AOT of 0.06 ± 0.02 over the Southern Ocean, which may be representative for clean marine conditions.

The AOT above the ocean is often much higher than 0.06 because of the presence of lofted aerosol layers caused by long-range transport of continental aerosols across the ocean. The MBL typically extends to 300–900m height [*Franke et al.* 2001; *Sugimoto et al.* 2001; *Groß et al.* 2011a]. The lofted continental aerosol can account for 30–60% of total marine AOT [*Ansmann et al.* 2001; *Franke et al.* 2003]. The long residence time of these lofted particles, their relevance for the evolution of clouds and precipitation, and their impact on radiative fluxes motivated a number of field campaigns in oceanic and coastal regimes (TRACE A [Fishman *et al.* 1996], ACE1 [*Bates et al.* 1998], TARFOX [*Russell et al.* 1999], ACE2 [*Russell and Heintzenberg*, 2000], INDOEX [*Ramanathan et al.* 2001], ACE Asia [*Huebert et al.* 2003], SAFARI [*Swap et al.* 2003], ABC [*Nakajima et al.* 2007], NAMMA [*Zipser et al.* 2009], and SAMUM [*Ansmann et al.* 2011a]). Ground-based and airborne lidars became more and more involved in these intensive field campaigns to provide vertically resolved aerosol observations in the MBL as well as in the free troposphere. Although complex campaigns provide detailed insights into the aerosol properties and the impact of aerosols on climatically relevant processes, these findings are representative for a certain area only. The global context remained an open issue. The situation improved significantly since 2006 when the CALIPSO mission began and the satellite-based lidar (CALIOP) started its operation [*Winker et al.* 2007]. This dual-wavelength backscatter lidar provides worldwide observations of lofted aerosol and cloud layers. *Kiliyanpilakkil and Meskhidze* [2011] determined a global mean AOT of 0.052 ± 0.038 for clean marine environments at 532nm. However, CALIPSO overpasses the same location at a velocity of 7000ms ^− 1^ each 16th day and thus provides only snapshot-like observations. Although CALIOP measurements allow to assess climatological mean aerosol profiles, a detailed study of regional aerosol features and evolution of aerosol layers is not possible. Furthermore, CALIOP is a backscatter lidar. Thus, aerosol type characterization is more difficult as well as the estimation of the extinction-to-backscatter ratio (lidar ratio) that is necessary to derive profiles of the extinction coefficient [*Sasano et al.* 1985]. Advanced ground-based lidars use various techniques (e.g., Raman scattering) to directly determine profiles of particle extinction coefficients and to allow a much more comprehensive aerosol characterization by means of measured lidar ratios [*Ansmann et al.* 1992; *Müller et al.* 2007].

Attempts to characterize the aerosol distribution over the ocean with shipborne lidars started in the 1990s. Single-wavelength micropulse lidars (MPL, [*Spinhirne et al.* 1995; *Campbell et al.* 2002]) were used during field campaigns like INDOEX [*Welton et al.* 2002], ACE2 [*Welton et al.* 2000], ACE-Asia [*Schmid et al.* 2003], SAFARI 2000 [*Campbell et al.* 2003], and during a meridional Atlantic cruise of the RV Ron Brown in the framework of AEROSOL99 [*Bates et al.* 2001]. Several cruises of the RV Mirai were used to perform dual-wavelength backscatter (532 and 1064nm) and polarization lidar measurements from 1999 to 2001 in the northern west Pacific region [*Sugimoto et al.* 2000; *Sugimoto et al.* 2001]. Aboard the RV Polarstern the Lidar Atmospheric Measurements Program (LAMP) backscatter lidar (355, 532, and 1064nm) observed the Mt.Pinatubo plume [*Philbrick et al.* 1992; *Stevens et al.* 1994] from October 1991 to January 1992. The Mobile Aerosol Raman Lidar (MARL) was deployed at Polarstern in 1996 and 2000. During the cruise in 2000, two Saharan dust plumes extending from 2–6km and from 2–4km height were observed [*Immler and Schrems*, 2003]. Recently, a single-backscatter and polarization lidar aboard the RV Marion Dufresne observed mixed layers of marine and biomass burning aerosol at the east coast of South Africa during the KAMASUTRA campaign [*Duflot et al.* 2011].

Even though shipborne lidar observations have been performed since about 20 years, mostly standard backscatter lidars were used for continuous operation. Tropospheric observations with Raman lidar or high-spectral-resolution lidar (HSRL) are much more suited for comprehensive aerosol studies, as mentioned. The use of such lidars is recommended by the GAW Aerosol Lidar Observation Network (GALION) working group [*Bösenberg and Hoff*, 2008].

On RV Polarstern, we deployed a multiwavelength Raman lidar with a polarization-sensitive receiver channel. The cruises were performed in the framework of the OCEANET project [*Macke et al.* 2010] in October–November of 2009, April–May of 2010, and October–November of 2010. In addition, extended lidar observations in the southern hemisphere for two 4 month campaigns at Punta Arenas (Chile, December 2009 to April 2010) and Stellenbosch (South Africa, December 2010 to April 2011) were conducted, while Polarstern was operated in Antarctica during southern-hemispheric summer time [*Kanitz et al.* 2011]. Further information about the experiment and the involved instruments is given in section 2. In section 3, the main findings are presented. Several case studies are discussed in detail to highlight the appreciable contribution of lofted aerosol layers to the overall aerosol load. A summary of the results and an outlook towards future investigations is given in section 4.

## 2. Experiment

**Figure 1:**
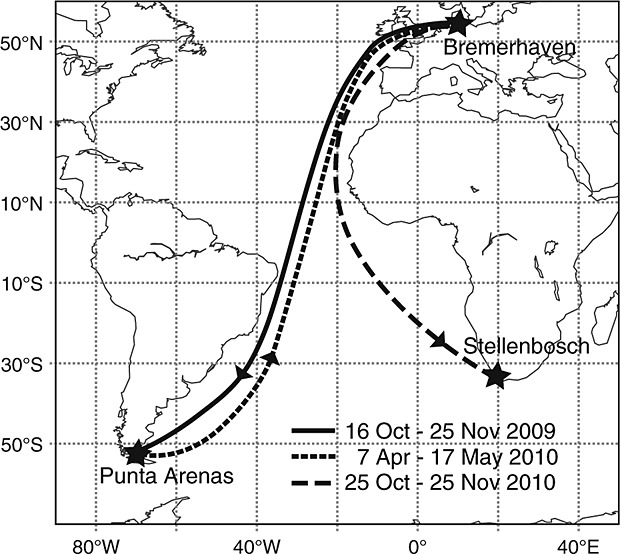
Map of the three meridional transatlantic cruises of Polarstern. The first cruise started at Bremerhaven and ended at Punta Arenas (solid line, fall 2009, ANT-XXVI/1). The second was from Punta Arenas back to Bremerhaven (dotted line, spring 2010, ANT-XXVI/4). For the third cruise, Polarstern departed from Bremerhaven and arrived at Cape Town (dashed line, fall 2010, ANT-XXVII/1). The lidar field sites of Punta Arenas and Stellenbosch in the southern hemisphere are indicated by stars.

The lidar observations were performed as part of the OCEANET project [*Macke et al.* 2010] 24/7, whenever the weather conditions were appropriate (groundswell < 6m, no precipitation). The goal of OCEANET is to continuously investigate the transfer of energy and material between ocean and atmosphere. The lidar was housed in a container aboard the German research vessel Polarstern, which is mostly operating under summer polar conditions either in the Arctic or in the Antarctic. The resulting transfer routes between Germany and either South America or South Africa provide an excellent opportunity to perform atmospheric and oceanic observations under tropical, subtropical, and midlatitudinal conditions in both hemispheres and to contrast atmospheric conditions and underlying processes [*Kanitz et al.* 2011]. The performed cruises and lidar locations are shown in Figure 1. The OCEANET lidar participated in a meridional cruise of Polarstern for the first time in the fall of 2009. The cruise went from Bremerhaven, Germany (16October 2009, ANT-XXVI/1) to Punta Arenas, Chile (25November 2009). Polarstern went on to Antarctica, while the lidar was unmounted from the container and deployed at Punta Arenas. The lidar was remounted onto Polarstern after a 4 month measurement campaign at the University of Magallanes to go from Punta Arenas (7April 2010, ANT-XXVI/4) back to Bremerhaven (17May 2010). The third cruise started in Bremerhaven (25October 2010, ANT-XXVII/1) towards Cape Town, South Africa (25November 2010) and a further campaign in Stellenbosch, South Africa from 2December 2010 to 13April 2011.

### 2.1. OCEANET Lidar Polly^XT^

The multiwavelength Raman and polarization lidar Polly ^XT^ [*Althausen et al.* 2009] enables the determination of vertical profiles of volume extinction coefficients of particles at 355 and 532nm, backscatter coefficients at 355, 532, and 1064nm, and the cross-polarized backscatter coefficient at 355nm with 30m vertical resolution. Hence, aerosol layers can be characterized by means of the lidar ratio at 355 and 532nm, which depends on the size, shape and chemistry of aerosol particles. For example, sea-salt consists of non-absorbing coarse mode particles with a low lidar ratio of about 20sr [*Groß et al.* 2011b]. In turn, smoke efficiently absorbs radiation and belongs to the fine mode. Thus, the lidar ratio is high with values > 70sr [*Tesche et al.* 2011]. In addition, the spectral dependence of the backscatter and extinction coefficient, the backscatter and extinction-related Ångström exponents [*Ansmann et al.* 2002] provide further information about the size of the particles [*Dubovik et al.* 2002]. The particle shape is characterized by the vertical profiles of the particle linear depolarization ratio at 355nm, too [*Freudenthaler et al*., 2009]. Further details of the data analysis methods are described by *Ansmann and Müller* [ 2005] and *Freudenthaler et al.* [ 2009]. Before OCEANET, Polly ^XT^ was operated in the Amazon basin from January to November 2008 [*Ansmann et al*., 2009, *Baars et al*., 2011, 2012].

The incomplete overlap between the footprint of the transmitted laser beams and the receiver field of view complicates the measurement of lidar return signals in the near field at heights below about 350–400m, which thus defines the minimum measurement height. The backscatter and extinction values in the MBL at heights below the minimum measurement height were set constant and equal to values determined for 400m height. In this way, it is assumed that the MBL is well mixed, which was found to be usually the case by comparing the photometer and lidar-derived AOTs for the marine boundary layer in the absence of lofted aerosol layers.

Because the boundary layer usually contains a much higher aerosol load than the free troposphere (see the discussion on Figure 9), the MBL top can be accurately determined with the wavelet method [*Baars et al.* 2008]. The MBL consists mainly of marine aerosol particles constantly emitted from the oceans. Therefore, the AOT of the MBL can be estimated by integrating the backscatter coefficient up to the MBL-top height and by multiplying this column backscatter value with a characteristic marine lidar ratio of 20sr [*Groß et al.* 2011b]. Because of possible contamination of the clean marine environment by continental aerosols [*Groß et al.* 2011a], the uncertainty in the MBL AOT estimation is about 20%.

### 2.2. Microtops Sun Photometer

In the framework of MAN shipborne Sun Photometer, measurements have been performed since 2004 [*Smirnov et al*., 2009]. Handheld Microtops photometers [*Morys et al.*, 2001] measure the spectral AOT at 340, 440, 500, 675, and 870nm. The AOT is defined as the column-integrated particle extinction coefficient (integrated over the vertical column). We use the cloud-screened and quality-assured level2 data over the Atlantic Ocean.

### 2.3. Supplementary Information

Air-mass transport analysis was partly based on the Flexible Lagrangian Particle Dispersion Model (FLEXPART) [Stohl *et al.* 2005]). It simulates long-range and mesoscale transport, as well as diffusion by resolved wind information and parameterized subgrid motions. The model uses archived meteorological data with a temporal resolution of 6h and a horizontal resolution of 1° × 1° provided by the Computational & Information Systems Laboratory that is managed by the National Center for Atmospheric Research. FLEXPART offers daily and cumulative snapshots of a density distribution of backward trajectories of 50,000 air parcels whose anchor points are distributed in the altitude range of interest above the observation site. For further approaches backward and forward trajectories were also calculated with the Hybrid Single-Particle Lagrangian Integrated Trajectory (HYSPLIT) model [*Draxler and Rolph*, 2003]. The local dust load in the area of Polarstern was estimated with the Dust Regional Atmospheric Model (DREAM) [*Pérez et al.* 2006]. Information about the meteorological conditions were obtained from the US National Weather Service's National Center for Environmental Prediction that is based on the Global Data Assimilation System (GDAS) [*Kanamitsu*, 1989]. For a horizontal grid of 1° × 1°, meteorological standard properties are stored in the GDAS1 data set, which contains 23 vertical layers up to about 30km height and is available every 3h.

## 3. Observations

We begin with an overview of the aerosol conditions over the Atlantic in terms of AOT as determined by a variety of sensors. This part is followed by the discussion of three case studies to provide a detailed insight into the vertical aerosol layering. Finally, latitude-dependent vertical aerosol profiles of backscatter and extinction coefficients are presented, and mean profiles for latitudinal belts in the midlatitudes and tropics are compared with special focus on differences between northern- and southern-hemispheric aerosol conditions.

### 3.1. Column-Integrated Optical Properties

**Figure 2:**
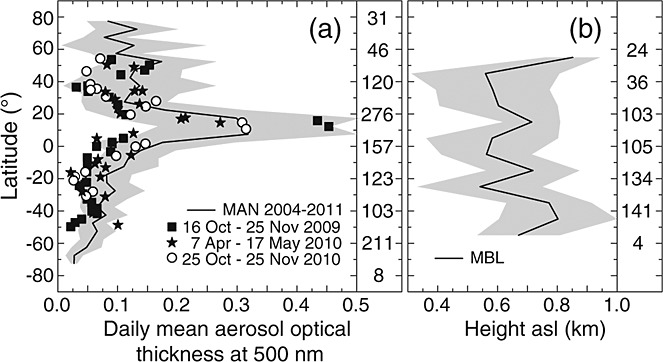
Latitudinal distribution of (a) mean aerosol optical thickness (solid line) and its standard deviation (shaded area) at 500nm in 5° latitude bins over the Atlantic from October 2004 to June 2011 as derived from MAN observations [*Smirnov et al*., 2009, extended time period]. Symbols indicate aerosol optical thickness (AOT) as derived from Sun photometer observations during the three Polarstern cruises. Latitudinal distribution of (b) maritime boundary layer height as derived from shipborne lidar observations. Right columns (in each of the two plots) show the number of observations within each of the 20° intervals.

Figure 2a shows the aerosol conditions over the Atlantic Ocean in terms of the mean AOT at 500nm and its standard deviation as derived from MAN observations between October 2004 and June 2011. Daily mean values of the AOT as determined with Sun photometer during the three performed cruises aboard Polarstern in 2009 and 2010 are shown in addition. Our 2009–2010 observations are in good agreement with the long-term (2004–2011) AOT data set. The highest values of AOT values were always found in the latitudinal belt between 0° to 20°N, i.e., in the outflow regime of Saharan dust. Maximum daily mean AOT was 0.46, 0.28, and 0.32 at 500nm in the fall of 2009, the spring of 2010, and the fall of 2010, respectively. In contrast, the smallest values of AOT of about 0.02 occurred in the southern hemisphere (fall 2009, 50°S, spring and fall 2010, around 20°S). The weak shift in the accumulation of our 2009–2010 AOT values towards lower values in the southern hemisphere compared to the 2004–2011 data set may result from the fact that the Polarstern cruises do not cover the full year, but only fall and spring seasons, and thus do not coincide with the biomass burning season in South America, taking place predominantly from July to October [*Baars et al.*, 2012]. The shown MBL heights in Figure 2b are derived from the lidar observations. In 78% out of all cases, the MBL top height was between 400 and 900m. This is in good agreement with measurements in the tropical Indian Ocean [*Franke et al.* 2001] and the tropical North Atlantic [*Groß et al.* 2011a]. Latitudinal and hemispheric differences in the vertical depth of the MBL were not found.

**Figure 3:**
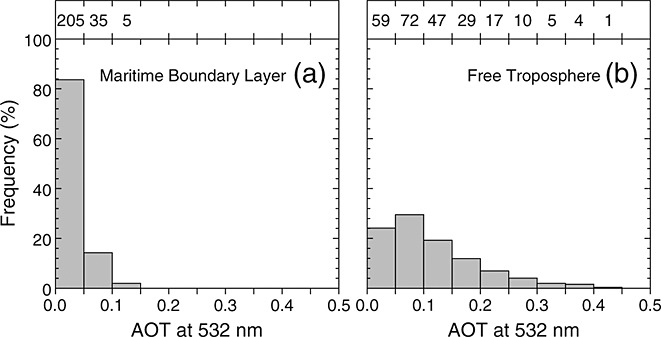
Frequency of occurrence of 532-nm AOT for (a) the marine boundary layer (MBL) and (b) the free troposphere (FT). The AOTs are calculated from respective column backscatter coefficients multiplied by a lidar ratio of 20sr (MBL) and 50sr (FT).

An important contribution of lidar to aerosol research is the potential to separate the optical properties of the MBL particles and free-tropospheric aerosols. In the free troposphere, particles can be transported over thousands of kilometers (from continent to continent, e.g., see *Mattis et al.* [2008] and *Baars et al.* [2011]) without removal by dry or wet deposition. Assuming a constant vertical extent of the MBL (Figure 2b) and a constant emission of marine aerosol particles from the ocean, advected aerosol layers in the free troposphere might be responsible for the increased AOTs in the northern tropics and the northern hemisphere. Figure 3 shows the frequency of occurrence of the AOT in the (a) MBL and the (b) free troposphere. Here, the strong contribution of lofted layers to the aerosol load in the atmospheric column above the Atlantic becomes obvious. The AOT values in the free troposphere are much higher than the MBL values. Whereas the AOT in the MBL is < 0.05 in more than 80% out of all cases, the free-tropospheric AOT is > 0.05 in almost 75% of the observations.

**Figure 4:**
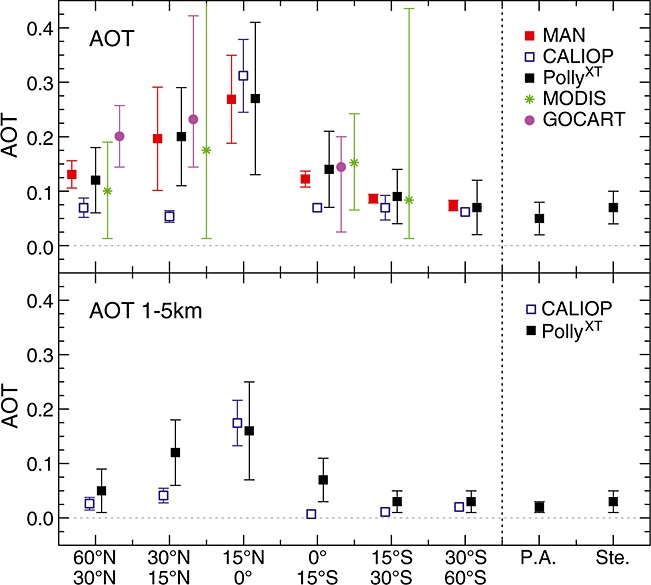
(Top) Mean AOT at 532nm for different latitudinal belts derived from Polly ^XT^ observations during three transatlantic cruises of Polarstern in 2009 and 2010 (black), from CALIOP overpasses (532nm, blue) during Polarstern observations, and from MAN Sun photometer observations (500nm) above the Atlantic from 2004–2011 (red), compared to MODIS satellite observations [*Kaufman et al.* 2005] (green), and AOT values modeled with GOCART (pink) [*Chin et al.* 2002]. Error bars show the range of measured AOTs (standard deviation for MAN, Polly ^XT^, CALIOP observations). Exact latitudinal ranges are given in the text. (Bottom) Mean AOT at 532nm from Polly ^XT^ observations and CALIOP observations for the height range from 1 to 5km height (free troposphere). P.A. and Ste. denote the measurement sites at Punta Arenas (53°S), Chile and Stellenbosch (34°S), South Africa.

Figure 4 (top) provides an overview of AOT observations, satellite retrievals, and results of a large-scale model. The AOTs derived in the framework of MAN (500nm), from Polly ^XT^ (532nm), and CALIOP (532nm) are given for the latitudinal belts of 60°– 30°N, 30°– 15°N, 15°– 0°N, 0°– 15°S, 15°– 30°S, and 30°– 60°S. An excellent agreement was found between the AOTs derived with the Sun photometers and from the Polly ^XT^ observations. The difference in the AOTs is only ± 0.02 and indicates the high accuracy of the lidar-derived AOT values. The mean AOT derived from CALIOP observations [*Anderson et al.* 2003; *Winker et al.* 2009] during 41 overpasses within a distance of 200km to Polarstern and within 1h related to the Polly ^XT^ observational period are lower than the respective ones from MAN Sun photometer and Polly ^XT^ measurements, except for the latitudinal belt from 15°N– 0°. Especially large discrepancies are found for the Saharan outflow region (15°– 30°N). The reason may be the low number of considered CALIOP observations so that the measurements are not representative. Figure 4(top) includes also AOT derived from MODIS retrievals (June–August 2002) for latitudinal belts from 60°–30°N, 30°– 5°N, 5°N– 20°S, and 20°– 30°S [*Kaufman et al.* 2005]. Results from the Global Ozone Chemistry Aerosol Radiation and Transport model (GOCART) [*Chin et al.* 2002] for latitudinal belts from 60°– 40°N, 30°– 10°N, and 0°– 20°S for January, April, July, and October 1997 are added to Figure 4(top) as well. The best agreement of the AOT observations is obtained for the southern tropics. GOCART seems to overestimate the AOT in the northern midlatitudes. Here, the AOT is higher than in the southern hemisphere, because the aerosol conditions are widely determined by anthropogenic pollution from North America, Europe, and East Asia, desert dust emissions in Africa, America, and Asia, and release of forest fire and other biomass burning smoke [*Mattis et al.* 2008]. In the northern tropics, lofted layers of continental aerosol from the outflow of the Saharan desert and Sahel region contribute highly to the aerosol concentration in the atmospheric column [*Kaufman et al.* 2005; *Ansmann et al.* 2011a]. In contrast, the aerosol conditions at the southern midlatitudes are widely controlled by MBL particles alone because of the absence of strong sources of anthropogenic haze and the comparably lower desert area. These results are corroborated by the AOT values determined from the Polly ^XT^ observations at the coastal field site of Punta Arenas (Chile, 53°S). Here, the lowest AOT was determined. At Stellenbosch (South Africa, 34°S) the AOT is comparable to the respective one for the latitudinal belt between 15° and 30°S.

Regarding free-tropospheric AOT, we can only compare Polly ^XT^ and CALIOP observations. The AOTs for the vertical column from 1–5km height are shown in Figure 4(bottom). AOTs in the free troposphere range from 0.01 (CALIOP, 0° to 15°S) to 0.17 (CALIOP, 15°N to 0°). The AOT in the height range up to 1km is 0.03–0.11 (40%–67% of the total AOT) as derived from Polly ^XT^ observations and 0.01–0.14 (22%–90%) according to CALIOP measurements. During ACE-2 and INDOEX, the boundary layer contributed, on average, 40%–70% to the total AOT [*Ansmann et al.* 2001; *Franke et al.* 2003]. *Groß et al.* [2011a] determined contributions from 5%–76% over Praia, Cape Verde during SAMUM–2.

When contrasting the AOT values for the latitudinal belts of 30°– 60°N and 30°– 60°S, we find values of 0.12 (0–5km height) and 0.05 (1–5km height) for the northern midlatitudes and 0.075 (0–5km) and 0.025 (1–5km) for the southern midlatitudes. Thus, the mean AOT over the remote ocean in the polluted northern midlatitudes is roughly a factor of 1.6, and in the free troposphere (1–5km), a factor of 2 higher than in the comparably clean southern midlatitudes. Regarding the indirect effect of aerosol particles, one can further expect that the cloud condensation nuclei (CCN) concentration over the remote ocean is a factor of > 2 higher at northern midlatitudes compared to southern midlatitudes when keeping the shift of the particle size distribution (fine mode) towards smaller radii with increasing contribution by pollution aerosol into account.

### 3.2. Profiling Aerosol Optical Properties

Three case studies of observed free-tropospheric aerosol layers are presented in the following. In doing so, we provide a deeper view into the occurrence of lofted aerosol layers, their dynamics, and the variability of particle optical properties with height. The first two measurements (31 October 2009 and 21 November 2009) were conducted during the first cruise and show pure dust plumes originating from the Saharan and Patagonian deserts. The third observation presents a complex mixture of Saharan desert dust, biomass burning aerosol, and marine aerosol.

**Figure 5:**
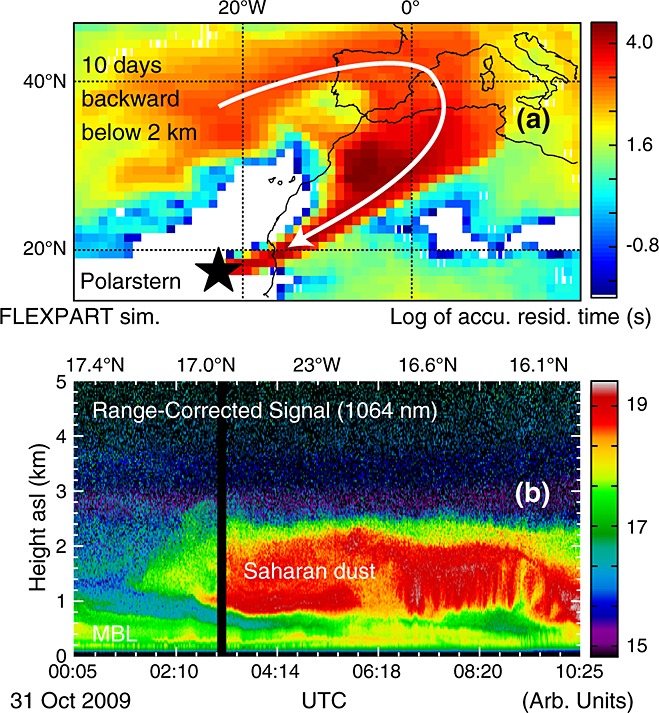
Map of (a) FLEXPART 10-day backward simulation of the observed air mass between 800–2500m height on 31October 2009 (3:20–5:20UTC) advected from the surface to 2000m height. The most probable air mass advection path is illustrated by a white arrow. The position of Polarstern is given by the star. (b) Height-time display of the range-corrected signal observed at 1064nm with the Polly ^XT^ lidar. A thick Saharan dust plume with lidar-derived AOT of 0.22 passed above the Polarstern.

During the first cruise in the fall of 2009 Polarstern passed the west coast of northern Africa at the end of October. On 31October 2009, a dense and extended Saharan dust plume was observed with Polly ^XT^ (Figure 5b). The aerosol-laden air mass was well separated from the MBL, which reached to heights of 500–800m. The base of the lofted layer was at about 900m, the top at about 2.5km height. After 10:00UTC the clear separation of the layer from the MBL disappeared (Figure 5b). Sun-photometer-derived AOT increased to 0.54 at 500nm (12:30UTC). The spectral dependence of the AOT in terms of the Ångström exponent was < 0.11 at 440/870nm indicating the presence and dominance of mineral dust [*Dubovik et al.* 2002].

FLEXPART 10-day backward simulations reveal the Saharan region as origin of the aerosol (dark red colors in Figure 5a). A high-pressure system off the west coast of Morocco advected air masses along the western flank of the Atlas Mountains (indicated by white arrow in Figure 5a). The Mediterranean air masses dried out, heated up and started to mobilize dust above the northern Sahara in Algeria. The optical properties of the aerosol layers are discussed below, after the introduction of the other two cases.

**Figure 6:**
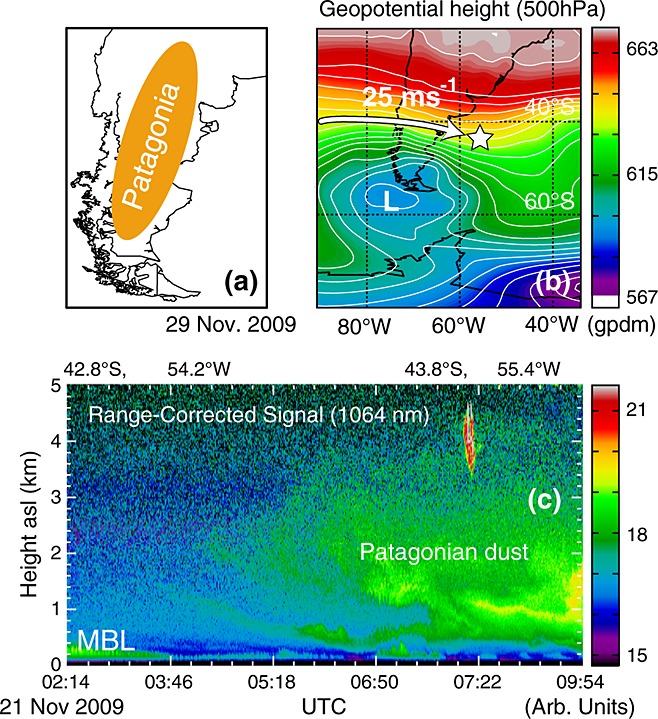
Map of (a) South America and location of Patagonia, (b) geopotential height (gpdm) at 500hPa and position of Polarstern (white star) on 21November 2009. White arrow indicates the air mass advection along the isohypses (height contours) with surface wind speeds of 25ms ^− 1^. (c) Lidar observation, performed on 21November 2009, of the arrival of a Patagonian dust plume with AOT around 0.03.

The second case deals with Patagonian mineral dust. During the first cruise, Polarstern changed its direction towards southwest when arriving at 20°S and reached the Strait of Magellan on 23November 2009 and finally Punta Arenas on 25November 2009. The vessel passed the Patagonian desert (Figure 6a) at a distance of about 1000km to the east on 21November 2009. Besides Australia, Patagonia is the only source of desert dust in the high southern latitudes. Its climate is dominated by westerly winds, which cross the Andean Mountains and lead to lee-effects in Patagonia. As a consequence, low amounts of precipitation and sparse vegetation consisting mainly of grass are characteristic for this area. Pressure gradients associated with cyclones to the south often produce winds exceeding 25ms ^− 1^ and dust emissions from the gravel desert surface by erosion [*Labraga*, 1994; *Gaiero et al.*, 2007; *Gassó et al.*, 2010]. This dust can be transported across the southern Atlantic [*Gassó and Stein*, 2007; *Li et al.* 2010; *Johnson et al.* 2011]. However, AOTs < 0.1 at 500nm were measured with Sun photometer aboard Polarstern (Figure 2, left) during the period from 13 to 23 November 2009 when the lidar continuously detected optically thin aerosol layers.

Figure 6c shows the height-time display of the lidar observation in the morning of 21November 2009. At the beginning, a shallow boundary layer with top at 350m height was found. After 4:00UTC, distinct lofted aerosol layers occurred. The layers extended up to 4km height at about 7:30UTC and decreased below 1km height until 11:30UTC (not shown). On that day, a cyclone of the Antarctic low-pressure belt was situated southwest of South America (denoted by L in Figure 6b). Strong westerly winds occurred in the latitudinal belt from 40°–45°S. According to the isohypse 10 min average wind speeds at 10m height were about 25ms ^− 1^ and likely triggered the dust uptake into the atmosphere and the transport to the Atlantic (as confirmed by FLEXPART calculation, not shown). AOTs derived in the framework of MAN ranged from about 0.06 at 12:40UTC on 20November to 0.03 on 21November 2009 and indicate background aerosol conditions [*Smirnov et al.* 2009].

**Figure 7:**
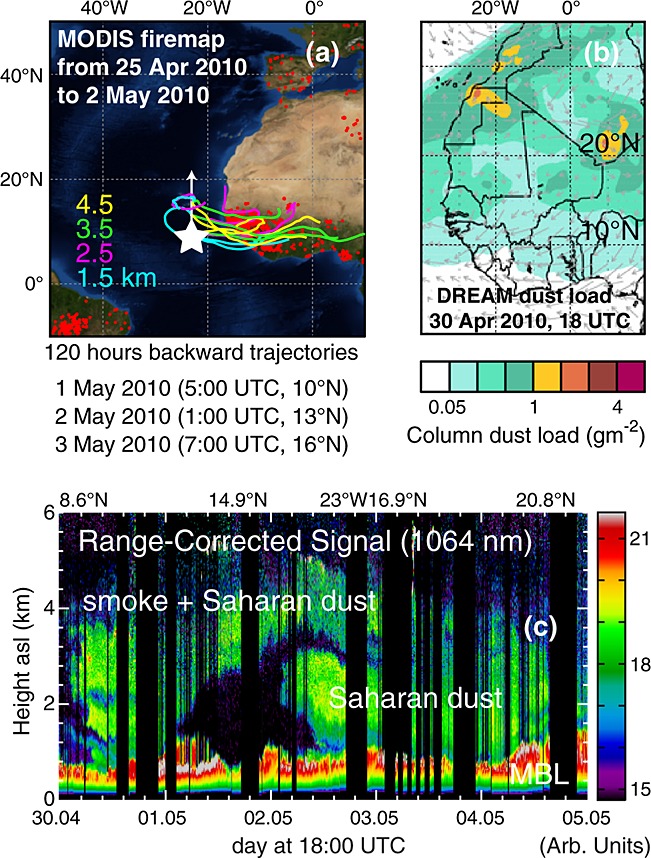
(a) Fire map derived from MODIS measurements and HYSPLIT 120-h backward trajectories for different arrival heights (indicated by different colors). Trajectory arrival times are given below the map. The white arrow shows the Polarstern cruise track from 30April to 5May 2010. (b) DREAM simulations of column dust load on 30April 2010, 18:00UTC. (c) Height-time display of the lidar backscatter signal from 30 April to 5 May 2010 showing complex aerosol layering of dust and smoke ( > 1000m) above the marine boundary layer (MBL). Lidar overlap effects (for heights < 400m) are not corrected.

The final observational example discussed here is taken from the lidar measurements between 30April 2010 and 5May 2010 and shows extended lofted aerosol layers occurring at the west coast of northern Africa from 8°– 21°N, 23°W (over a north-south distance of more than 1400km) over several days (Figure 7c). The photometer-derived 500-nm AOTs showed maximum values of 0.3 on 2May 2010 and Ångström exponents dropped to values < 0.3. As shown in Figure 7a Polarstern was located more than 1000km west of an extended area of biomass-burning activity in western Africa on 30April 2010, 18:00UTC. Marine aerosols most probably prevailed in the MBL, whereas the lofted aerosol layer contained considerable amounts of biomass-burning smoke and dust. HYSPLIT backward trajectories are presented for arrival heights above the Polarstern from 1.5 to 4.5km in Figure 7a. Only on 3May 2010, the backward trajectories in the range from 1.5 to 2.5km height do not cross the area of biomass-burning activity. DREAM calculations given in Figure 7b show an increased dust concentration north of 8 ^∘^ N and a further increase at about 13°N, which is in agreement with the lidar observation (Figure 7c).

**Figure 8:**
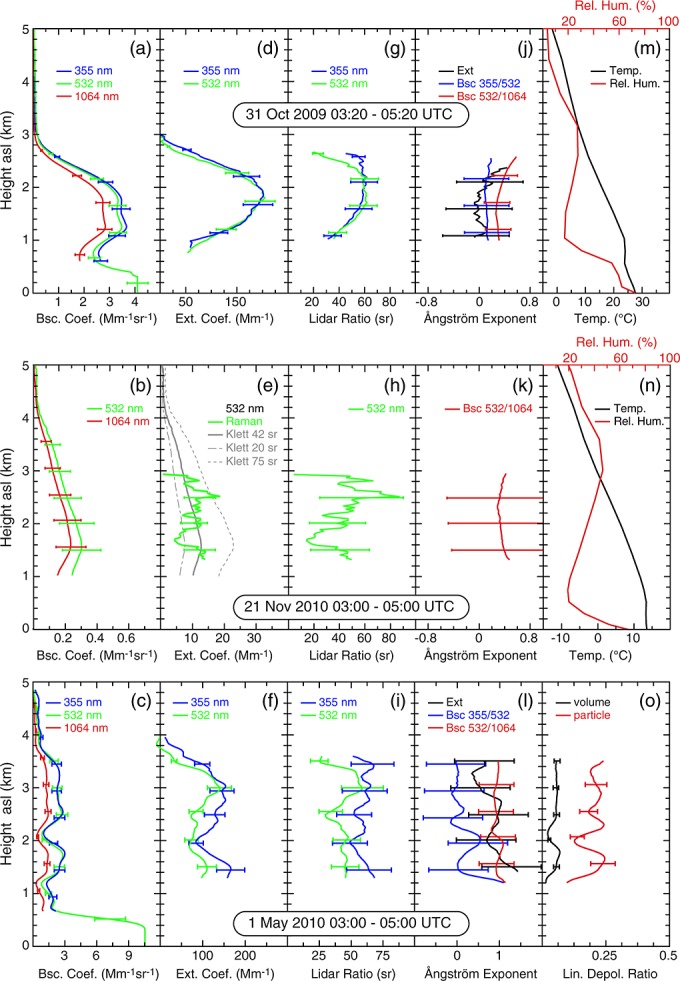
(a–c) Profiles of particle backscatter coefficient, (d–f) extinction coefficient, (g–i) lidar ratio, and (j–l) Ångström exponent derived from Polly ^XT^ observations on (top) 31October 2009, 3:20–5:20UTC, (center) 21November 2009, 6:00–8:00UTC, and (bottom) 1May 2010, 3:00–5:00UTC. (m, n) Temperature and relative-humidity profiles for 31October and 21November 2009 from GDAS1. (o) The linear depolarization ratio from Polly ^XT^ observation for 1May 2010.

Figure 8 presents an overview of the particle optical properties of the three cases. Profiles of the particle backscatter coefficient (Figures 8a– 8c), extinction coefficient (Figures 8d– 8f), corresponding lidar ratios (Figures 8g– 8i), and Ångström exponents (Figures 8j– 8l) are shown. In addition, for the Saharan and Patagonian dust plumes, observed profiles of temperature and relative humidity from GDAS1 are given in Figures 8m and 8n, respectively.

The Saharan dust plume extended from about 800–3000m height. Relative humidity in the plume was only 20–30%. The data analysis for the time period from 3:20 to 5:20UTC reveals wavelength-independent particle backscatter and extinction coefficients at 355 and 532nm (Figures 8a and 8d) for the height range from 800–3000m. The low extinction and backscatter-related Ångström exponents of 0.09 ± 0.16 and 0.12 ± 0.02, respectively, at heights from 1.1 to 2.4km clearly indicate the presence of Saharan dust. The backscatter coefficient at 1064nm (red profile in Figure 8a) was about 20% lower than at 532nm, corresponding to an Ångström exponent of 0.33 ± 0.06 (red curve in Figure 8j). This is in good agreement with SAMUM-1 and SAMUM-2 observations [*Tesche et al*., 2009, 2011; *Groß et al*., 2011b]. Mean desert-dust lidar ratios of 50–60sr (Figure 8g) in the center of the dust layer at both wavelengths (355 and 532nm) are also in very good agreement with the SAMUM results. The AOT for dust was 0.22, which was about two-thirds of the total AOT.

The in-depth analysis of the lidar observation concerning the Patagonian dust plume was difficult because of the rather low AOT of the lofted aerosol plume of 0.02–0.03. Taking the high surface wind speeds above the Patagonian desert into consideration (see Figure 6b), the low AOT suggests less available particulate material in the Patagonian desert that can be lifted into the atmosphere than in the Saharan desert. The vertical smoothing length was set to 750m to reduce signal noise, and for technical reasons, the signal profiles could be used only above 1000m height. With respect to the signal-to-noise ratio, the uncertainty is estimated to be 40%. The profiles of particle backscatter coefficient (Figure 8b) show an increased aerosol loading up to 4km height. According to the temperature profile available from GDAS1 in Figure 8n, the MBL reached up to 500m, and the base of the lofted layer was at 1000m. The 532-nm profile of the extinction coefficient determined with the Raman method is given in Figure 8e by the green curve. The extinction coefficient was rather low with values around 10Mm ^− 1^. A mean lidar ratio of 42 ± 17sr was calculated, which is in the range of dust-related lidar ratios found in the Middle East (42.6sr and 45sr) and Kanpur, India (43.8sr) [*Müller et al.* 2001; *Schuster e al.* 2012]. In addition, the profile of the extinction coefficient at 532nm was reproduced by analyzing the 532-nm elastic backscatter signals with the so-called Klett method [*Klett*, 1981]. In this approach, a height-constant lidar ratio has to be assumed. We used lidar ratios of 42sr (suggested for Patagonian desert dust), 20sr (indicating pure marine aerosol [*Groß et al.* 2011b]), and 75sr (indicating smoke [*Tesche et al.* 2011]). These approximated profiles of extinction lead to AOTs of 0.03, 0.02, and 0.05 for the plume, which are much smaller than AOTs of the observed Saharan dust event presented before. A low backscatter-related Ångström exponent of 0.4 ± 0.1 at 532 and 1064nm (see Figure 8k) indicates the presence of coarse-mode particles.

Another hint for the presence of large dust particles are the ice virgae shown at the top of the aerosol layer at about 7:15UTC in Figure 6c. Dust particles are known to be favorable ice nuclei and can trigger heterogeneous ice nucleation at comparably high temperatures [*Seifert* *et al.* 2010]. In the present case, the ice nucleation was initiated at cloud top temperatures of roughly − 10°C.

The bottom panel of Figure 8 provides the profiles of the particle backscatter coefficient (Figure 8c), extinction coefficient (Figure 8f), and corresponding lidar ratios (Figure 8i), Ångström exponents (Figure 8l), and linear depolarization ratios (Figure 8o), which were determined from lidar measurements on 1 May 2010. Two lofted layers show up in the profiles of the particle backscatter coefficient (Figure 8c). The lower layer extends from 1.3 to 1.9km height, the upper layer extends from 2.4 to 3.5km height. AOTs of 0.15 and 0.11 (355 and 532nm) for the upper layer, and 0.08 and 0.05 for the lower layer are derived from the extinction profiles. The lidar ratios are, on average, 61 ± 4sr and 45 ± 11sr for the upper layer and 59 ± 5sr and 45 ± 2sr for the lower layer at 355 and 532nm, respectively. For the extinction-related (355/532) and backscatter-related (355/532 and 532/1064nm) Ångström exponents of the upper layer (lower layer), we obtained values of 0.7 ± 0.3 ( 1.1 ± 0.2), 0.0 ± 0.1 ( 0.2 ± 0.1), and 0.9 ± 0.0 ( 1.0 ± 0.1), respectively. The particle linear depolarization ratio is, on average, 21% ± 2% in the upper layer and 22% ± 3% in the lower layer. The layer mean lidar ratio at 355nm is higher than the typical value for pure dust (55sr), and the particle linear depolarization ratio is smaller than the pure-dust values of > 26% [*Groß et al.* 2011b]. Together with the comparably high Ångström exponents, these values indicate a mixture of biomass-burning smoke aerosol and dust [*Tesche et al.* 2011].

A new lofted aerosol layer was observed on 3May 2010 (Figure 7c). The layer extended from about 1.5 to 3.0km height. The mean AOT of this layer was around 0.11. The extinction-related Ångström exponent was around 0.4 ± 0.4, and the particle linear depolarization ratio increased to values of 27% ± 2%. By assuming a particle linear depolarization ratio of 25%–29% for pure dust, and of 3%–5% for smoke, the first observed lofted layers showed a smoke fraction of 10%–30% when applying the dust/smoke separation method of *Tesche et al.* [2009]). The second lofted layer contained only dust.

### 3.3. Latitudinal Mean Particle Backscatter and Extinction Profiles

**Figure 9:**
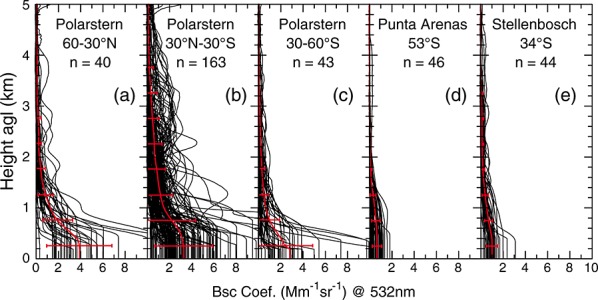
Cloud-screened lidar profiles of the backscatter coefficient at 532nm in the (a) northern midlatitudes, (b) tropics, and (c) southern midlatitudes as determined during three transatlantic cruises. Four-month lidar observations at (d) Punta Arenas and (e) Stellenbosch are given for comparison. Red profiles show the mean profiles. Error bars indicate the mean standard deviation caused by atmospheric variability.

For a statistical analysis 246 cloud-free measurement periods (about 260h) were selected. All single profiles of the particle backscatter coefficients at 532nm for the northern midlatitudes (60°–30°N), the tropics ( 30°N– 30°S), and the southern midlatitudes (30°–60°S) are presented in Figures 9a– 9c. Averaged profiles of the backscatter coefficients in each region are shown by red lines. The error bars indicate the mean standard deviation. The profiles of the particle backscatter coefficient over Punta Arenas and Stellenbosch are added in Figures 9d– 9e. Below the MBL top height, well-mixed homogeneous particle conditions are assumed. Thus, the particle backscatter coefficient is set constant from the minimum measurement height down to the ground. Signals are smoothed with 330m vertical window length for the analysis.

The distribution of the 532-nm backscatter coefficient profiles show that lofted aerosol layers occur most frequently in the tropics (Figure 9b), and more often in the northern midlatitudes (Figure 9a) than in the southern midlatitudes (Figure 9c) over the Atlantic. Note the comparably clean conditions over the field sites of Punta Arenas and Stellenbosch (Figures 9d– 9e).

**Figure 10:**
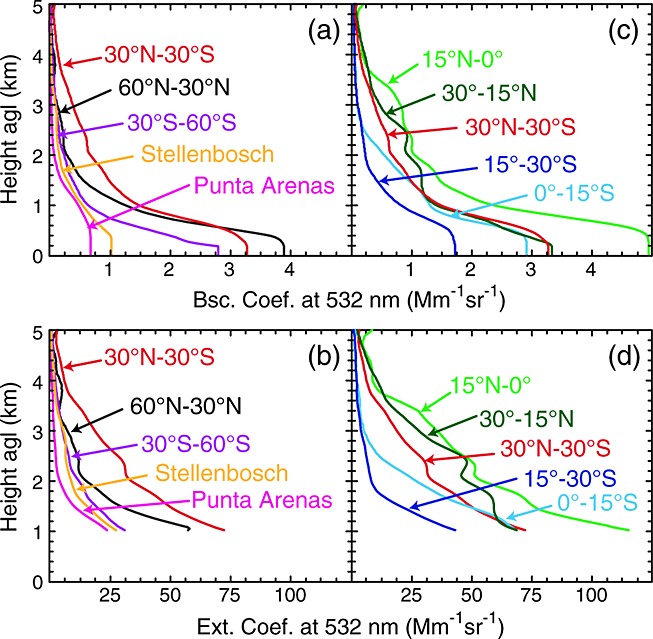
Mean particle backscatter and extinction coefficients at 532nm (a–b) for the northern midlatitudes (60°–30°N), tropics (30°N– 30°S), southern midlatitudes (30°–60°S), Punta Arenas, Stellenbosch, and (c–d) for five latitudinal belts of the tropics.

An overview of the mean profiles of the backscatter coefficient is given in Figure 10a. Figure 10b shows the corresponding profiles of the extinction coefficient above 1km height. The extinction values are estimated from the backscatter coefficients in Figure 10a by multiplying them with a lidar ratio of 50sr. The extinction coefficient in the tropics is a factor of two higher than in the northern midlatitudes in the height range from 2–4.5km. In agreement with the observed AOT difference (a factor of 1.5–2 higher values at northern midlatitudes), the extinction coefficient in the northern midlatitudes is higher than in the southern midlatitudes. The difference in the optical properties is visible up to about 3km height.

The extinction coefficients at the most southern measurement site of Punta Arenas are the lowest observed and are close to zero at already 2km height. The aerosol profiles at Punta Arenas (Figure 9d), are almost completely determined by marine sources. In comparison to the shipborne observations in the northern midlatitudes or previous lidar observations in the frame of the European Aerosol Lidar Network (EARLINET) [*Mattis et al.* 2008] the occurrence of free-tropospheric aerosol layers at Punta Arenas in the southern midlatitudes is rather low because of the absence of continental aerosol sources. At Stellenbosch lofted layers of smoke and soil dust from local sources are usually observed up to about 4km height (Figure 9e).

Mean profiles of the backscatter and extinction coefficient for 30°– 15°N, 15°N– 0°, 0°– 15°S, and 15°– 30°S are presented in Figures 10c–10d. The highest aerosol concentration is found in the latitudinal belt from 15°N– 0° (3.3 times higher than the northern midlatitudes shown in Figure 10a), followed by 30°– 15°N (2.5 times higher), 0°– 15°S (96%), and 15°– 30°S (72%). *Tesche* [2011] determined mean particle backscatter coefficients at 532nm of 0.4 and 1.2Mm ^− 1^sr ^− 1^ in the main lofted layers from 1.5 to 4km height at Cape Verde during winter (15January to 14February 2008) and summer (24May to 16June 2008), respectively. In the same height range, we found mean particle backscatter coefficients of 0.8 and 0.65Mm ^− 1^sr ^− 1^ for the latitudinal belts from 15°N– 0° and from 30°–15°N. The difference between the aerosol conditions in the northern and southern tropics is most pronounced in the height range from 2 to 4km (Figure 10d). While in the northern tropics, the mean extinction coefficient is up to 50Mm ^− 1^, the extinction coefficient in the southern tropics decreases down to nearly zero at about 3km height.

## 4. Conclusion and Outlook

The vertical distribution of aerosols above the North and South Atlantic was analyzed with shipborne multiwavelength Raman/polarization lidar. The presented results were based on lidar measurements in the framework of the OCEANET project aboard the research vessel Polarstern during three transfer cruises over the Atlantic Ocean, between Germany and either South America or South Africa. Each of the meridional cruises covered a distance of about 10,000–14,000km and corresponding periods of 4–6weeks. Layers of Saharan dust partly mixed with biomass-burning smoke were observed at the west coast of North Africa. Based on the lidar depolarization measurements, a biomass-burning smoke contribution to light backscattering of up to 30% was found in an observed mixed layer. For the first time, plumes of Patagonian desert dust were characterized with lidar. The shipborne lidar observations revealed a 532–nm lidar ratio of 42 ± 17sr. Although the wind speed above the Patagonian desert was high with ≈ 25ms ^− 1^, the AOT was low with 0.02–0.03.

The statistical analysis revealed that the marine boundary layer extended up to about 0.4–0.9km height and lofted layers were frequently observed up to 4–5km height. The MBL AOT for 532nm accumulated around 0.05. The free-tropospheric aerosol layers of continental origin significantly contribute to the observed total AOT and frequently cause total AOTs > 0.1–0.15. Distinct differences in the meridional distribution of aerosol profiles were found. The highest optical thickness was determined in the northern tropics (0.27), mainly in the outflow region of Saharan dust. South of the equator, a sharp decrease in the amount of free-tropospheric aerosol in the tropics was found. The strong north-to-south difference in the aerosol load also holds for the midlatitudes. It was found that the AOT and extinction coefficients for the vertical column from 0–5km (total aerosol column) and 1–5km height (lofted aerosol above the marine boundary layer) at northern midlatitudes are a factor of 1.5–2 higher than at respective southern midlatitudes, and even a factor of 2.5 higher than at the clean marine southern-hemispheric site of Punta Arenas. The strong contrast is confined to the lowermost 3km of the atmosphere. The higher aerosol concentrations over remote ocean sites in the northern midlatitudes might not only partly reflect the higher impact of desert areas but also clearly indicate the distinct contribution from anthropogenic emissions.

The meridional transatlantic cruises of Polarstern cover only the spring and fall seasons. Thus, our observations do not capture all the intra- and interannual variability of the aerosols in the atmosphere. However, we found promising agreement to long-term shipborne Sun photometer measurements and spaceborne observations. The lidar measurements will be continued aboard Polarstern for the next 5–10 years. The increasing data set will enable a more detailed latitudinal investigation of tropospheric aerosols, and serve as a unique ground-based validation of height-resolved spaceborne observations over the Atlantic. In the poorly investigated southern hemisphere, the shipborne lidar measurements are even more valuable, because measurement stations on shore are limited [*Antuña et al.* 2012]. Synergistic measurements of microwave radiometer, and pyranometer aboard Polarstern will improve the understanding of the global influence of aerosols and aerosol-cloud interactions.
